# Differential Coping Strategies in Response to Salinity Challenge in Olive Flounder

**DOI:** 10.3389/fphys.2019.01378

**Published:** 2019-11-06

**Authors:** Junjia Zeng, Neill A. Herbert, Weiqun Lu

**Affiliations:** ^1^National Demonstration Center for Experimental Fisheries Science Education, Shanghai Ocean University, Shanghai, China; ^2^Key Laboratory of Exploration and Utilization of Aquatic Genetic Resources, Ministry of Education, Shanghai Ocean University, Shanghai, China; ^3^International Research Center for Marine Biosciences, Ministry of Science and Technology, Shanghai Ocean University, Shanghai, China; ^4^Leigh Marine Laboratory, The University of Auckland, Warkworth, New Zealand

**Keywords:** flounder, salinity challenge, coping strategy, NKA-α1, NHE-3-like

## Abstract

To examine how different fish coping strategies respond to salinity challenge, olive flounder (*Paralichthys olivaceus*) with active coping style (AC) and passive coping style (PC) were transferred from seawater (SW) to freshwater (FW) and their behavior and physiology were analyzed. Different behavioral coping strategies, in terms of escape and feeding tendencies, were confirmed in AC and PC fish without FW exposure. Differences in swimming distance between AC and PC flounder were then assessed after 1 and 2 days of FW transfer. Plasma parameters and branchial gene expression were also determined 2, 5, 8, and 14 days after transfer, with comparisons between AC and PC fish and against a SW-acclimated control group. The results showed that: (1) PC flounder exhibited a significant reduction in swimming activity, while AC flounder significantly increased locomotion 2 days after transfer. (2) The plasma osmolality and plasma ionic (Na^+^ and Cl^−^) concentration of FW-acclimated PC flounder declined in a continuous fashion over time but this contrasted against the plasma parameters of AC flounder which fluctuated below the baseline level of a SW-acclimated control group. (3) The expression of NKA-α1 and NHE-3-like mRNA in PC flounder gill increased significantly from 5 days, but the expression of these two genes in AC flounder only increased after 8 days of transfer. (4) There were no remarkable differences observed in Rhcg expressions between AC and PC flounder. This study indicates for the first time that PC flounder adopt a “freeze-passive tolerance” strategy while AC flounder adopt a “flight-active resistance” defense strategy in response to salinity challenge.

## Introduction

In animals, including fish, coping style is defined as a consistent individual response (CIR) to environmental challenge that is maintained over time and/or across contexts (Castanheira et al., 2017). Moreover, the CIRs of fish in response to environmental challenges are associated with behavioral and physiological differences (Overli et al., 2007), and have increasingly been recognized as adaptive variation (Castanheira et al., 2017). Differences in osmoregulatory ability and behavioral patterns (e.g., feeding attempts, captured and uncaptured) also exist in teleosts with different coping styles (Sloman et al., 2003, 2004, 2008; Louison et al., 2017). In common with the rainbow trout *Oncorhynchus mykiss* (Frost et al., 2007), active coping (AC) individuals and passive coping (PC) individuals are observed in olive flounder *Paralichthys olivaceus* (Rupia et al., 2016). Compared to PC flounder, AC flounder show differences in response to simulated capture, feed propensity and metabolic rate (Rupia et al., 2016). For example, during acute stress, passive coping individuals adopt a passive “freeze-hide” strategy by reducing their oxygen consumption rates and remaining immobile whereas active coping individuals adopt an active “fight-flight” defense strategy by increasing their rates of respiration and activity levels (Rupia et al., 2016).

In fish, marine teleosts are hypoosmotic to their environment, which requires them to ingest seawater (SW) to offset the osmotic loss of water and excrete NaCl to reduce the excessive diffusive gain of salt. In contrast, freshwater teleosts are hyperosmotic to their environment which requires that they extract NaCl from their surrounding freshwater (Hwang and Lee, 2007; Evans, 2008). Euryhaline fish such as olive flounder, encounter complicated salinity changes during their spawning migration (Bolasina et al., 2007) and need to maintain body fluid homeostasis through sophisticated mechanisms of ionic and osmotic regulation (Kang et al., 2008). Although Na^+^ and Cl^−^ are the dominant ions in plasma, euryhaline marine teleosts in freshwater have lower salt content compared to euryhaline fish in seawater (Evans, 2008). The fish gill is an essential organ that plays a dominant role in osmotic and ionic regulation, mainly due to the presence of chloride cells that perform ion transport (Evans et al., 2005; Hwang, 2009). In freshwater (FW), ionocytes actively transport Na^+^ and Cl^−^ from the external aquatic environment to compensate the passive loss of ions in the urine and diffusion from the permeable surfaces of the body and gill (Hsu et al., 2014). Na^+^-K^+^-ATPase (NKA) as a primary Na^+^ transporter has been localized to the basolateral region of ionocytes, and provides an electrochemical gradient for active ion movements (e.g., Na^+^ and Cl^−^) across the branchial epithelia to maintain intracellular homeostasis (Hwang et al., 2011). It had also been demonstrated that salinity challenge increases the abundance of NKA-α-subunit mRNA (Scott et al., 2004; Hwang and Lee, 2007) and most euryhaline teleosts exhibit adaptive changes in NKA activity in response to salinity challenge (Feng et al., 2002; Weng et al., 2002; Hwang and Lee, 2007). Moreover, another Na^+^/H^+^ exchanger (NHE3) and a Rh glycoprotein (Rhcg) are both suggested to be involved in the model for ammonium-dependent Na^+^ uptake: NHE3 down the extracellular apical H^+^ gradient created by ammonia diffusion of Rhcg, simultaneously extract sodium from environment to compensate the Na^+^ uptake (Esaki et al., 2007; Yan et al., 2007; Hwang et al., 2011). Therefore, based on the accumulating evidence, a model of NaCl movement across the gill of euryhaline teleosts has been established (Hwang and Lee, 2007). Salinity challenge is common for flounder as they inhabit shallow coastal embayments and estuaries that fluctuate in salinity level. An increase in the intensity and frequency of extreme events is predicted to occur as a result of climate change. In the estuary, hurricanes, flooding and heavy rainfall can cause dramatic changes in water conditions, especially salinity. Flounder is therefore forced to face with unusual salinity challenge (Bailey and Secor, 2016; Williams et al., 2017). Flounder also encounter complicated salinity changes during their spawning migration. The ability of individuals to “cope” with such a challenge will therefore influence their ecology and, hence, their vulnerability to capture. An understanding of individual coping strategy is therefore warranted.

A link between coping style and osmoregulation in teleost fishes has been reported previously (Sloman et al., 2003). But there is still a shortage of information that links the behavior and physiology of euryhaline fish with different coping styles to salinity stress. For example, do these fish adopt different behavioral and physiological coping strategies under salinity stress? In order to answer this question and advance knowledge in the field, our study aims to provide more detail regarding the behavioral and physiological mechanisms by which olive flounder, with different active and passive coping strategies, contend with salinity challenge. To achieve this aim, the following parameters were measured in euryhaline flounder with different coping styles transferred from SW to FW: (1) swimming activity, behavioral feeding and escape response, (2) dynamic change in plasma osmolality and ionic concentration (Na^+^ and Cl^−^), and (3) the branchial gene expression levels of NKA-α1, NHE-3-like, and Rhcg.

## Materials and Methods

### Experimental Fish

To reduce the potential influence of sexual dimorphism in fish (Yuan et al., 2017), gynogenetic olive flounder (body weights: 500 ± 50 g) were produced and reared in SW (30‰) recirculating aquaculture systems at the Central Experimental Station of Chinese Academy of Fisheries Sciences in November 2018 (Beidaihe, Hebei, China). About 80 active coping (AC) individuals and 80 passive coping (PC) individuals were selected from PC and AC broodstock screened according to behavioral tests developed by Rupia et al. (2016). Gynogenetic olive flounder of each coping style were then distributed evenly into each of the 20 holding tanks (500 L) and maintained for 2 weeks with filtered flow-through SW at a salinity level of 30‰. Water temperature was maintained at 18 ± 1°C, with a constant 12 h light/12 h dark cycle (8:00 am–20:00 pm). The mean light intensity, measured centrally at the bottom of each tank, was approximately 40 lux. Fish were starved throughout the experiment to ensure that differential rates of feeding did not influence the results of the study (Yuan et al., 2017).

### Behavioral Test to Confirm the Presence of Passive Coping Style and Active Coping Style Strategies

To confirm the behavioral type of each AC and PC family, gynogenetic olive flounder were selected at random from the main holding tanks and isolated individually in smaller holding tanks (size 300 L). About 48 experimental fish (*n* = 24 for AC and *n* = 24 for PC) then had their behavior screened for two different aspects of coping. Propensity to feed and air exposure are commonly used in fish studies to distinguish behavioral types (Rupia et al., 2016), and these two behavioral tests were also used in this experiment for confirmatory purposes. In the first feeding behavior test, a piece of raw fish was attached to a rod with a thin piece of line and held approximately 10 cm above the head of an individual fish for 2 min. The time taken to initiate the first feeding strike in 2 min was recorded as “feeding tendency.” To ensure that the fish did not actually feed during the trial, the food item was pulled out of the water if the fish approached it within about half a body length. In the second test, an individual was removed from the holding tank in a net and held in air for 2 min. The time taken to initiate the first escape attempt was recorded as “escape tendency” and the total number of escape attempts was recorded as “escape attempt,” defined as the number of body movements that resulted in an elevation of the body from the net (Rupia et al., 2016).

### The Behavioral Response of Active and Passive Coping Styles to Acute Salinity Challenge

Olive flounder with confirmed active coping style (AC, *n* = 8) and confirmed passive coping style (PC, *n* = 8) were selected at random and placed individually into 16 opaque 100 L seawater tanks (*n* = 1 per tank; salinity = 30‰; water depth = 20 cm). Individuals could not see each other in these boxes. Cameras were mounted centrally above the tanks and trials began at 8:00 am. After recording the behavior of fish for 2 days, the flounder were transferred from 30‰ seawater (control group) to different set of 16 freshwater tanks at 3‰, with behavior monitored for a further 2 days. The swimming distance of flounder from video recordings was then computed using specialized behavioral software (Big Brother version 3, ACTIMETRICS, Wilmette, Illinois, USA).

### The Ionic and Gene Expression Response of Active and Passive Coping Styles to Salinity Challenge

Olive flounder with confirmed active coping style (AC, *n* = 48) and confirmed passive coping style (PC, *n* = 48) were netted and randomly divided into mixed coping groups with direct transference to four seawater (SW, 30‰, control group) tanks or four freshwater (FW, 3‰) tanks (*n* = 6 for each coping style in each tank). SW-acclimated and FW-transferred fish were then sampled during daylight hours 2, 5, 8, and 14 days after initial transfer. All fish in each tank were rapidly netted at each time point tank and blood samples (3–5 ml) were collected within 90 s using a heparinized needle and syringe (200 U/ml heparin, Sigma) by caudal venipuncture. Blood was then aliquoted into ammonium-heparinized tubes (200 U/ml, Sigma) and plasma separated by centrifugation for 5 min at 13,000 rpm and stored at −80°C for the subsequent measurement of plasma osmolality and plasma ionic concentrations (Na^+^ and Cl^−^). Fish were then killed humanely by severing the spinal cord and destruction of the brain. The gill was removed and stored at −80°C for the later analysis of branchial gene expression.

Plasma osmolality was measured using a vapor pressure osmometer (Wescor 5200, Logan, UT, USA). Plasma Na^+^ and Cl^−^ concentration was measured using DIONEX ICS-1500 for cation and DIONEX ICS-900 for anion (Thermo, scientific, USA).

Total RNA was extracted from the gills using RNAiso Plus (TaKaRa, Japan). About 500 ng of total RNA was reverse-transcribed into cDNA using PrimeScript™ RT reagent (Takara, Dalian, China) following the standard protocol. NKA-α1, NHE-3-like and Rhcg gene expressions were collected on ABI 7500 (Applied Biosystems, Carlsbad, CA) with SYBR Premix Ex Taq™ (Takara, Dalian, China). β-actin as the housekeeping gene, was using to normalized branchial gene expression levels. The following PCR conditions were used: 10 min at 95°C, followed by 36 cycles of 95°C for 10 s, 30 s at 60°C. Gene sequences are obtained from NCBI and all the primers designed by NCBI primer blast. The forward and reverse primers span an exon-exon junction. All the primers are tested and showed to be viable and specific. The primer sets for the quantitative RT-PCR are shown in Table 1.

**Table 1:** Primer sequences used for real-time PCR amplifications.

Gene	GenBank	Primer(5′-3′)
β-actin	HQ386788.1	F: GGAAATCGTGCGTGACATTAAG R: CCTCTGGACAACGGAACCTCT
NKA-α1	XM_020104090.1	F: CATCAGCATCGCTTACGG R: GGGAAGGCACAGAACCAC
NHE-3-like	XM_020108892.1	F: CAGAGCAGGAGCTGGAAT R: GACAGGAGTGTCGGCAAG
Rhcg	XM_020078180.1	F: TTTGTGACAGACTGGAGGTG R: TCTAACGGCAACAAGGGA

### Statistical Analysis

The 2^−ΔΔ Ct^ method was used to analyze the quantitative RT-PCR data (Yuan et al., 2017), and the expression level in all plots of each gene are presented as the relative change in values from SW-acclimated AC at 14 days, which was set at 1. All statistical analysis was carried out using SPSS Statistics 20 or XLSTAT@2014. The normality and homogeneity of variance of data were tested using Shapiro-Wilk’s test and Levene’s test respectively. A principal component analysis (PCA) according to behavioral test, examining feeding and escape behavior was performed in XLSTAT@2014. Two-way analysis of variance (ANOVA) with Tukey *post hoc* comparisons were used to determine the effect of coping style and salinity (SW versus FW) on swimming activity at day 1 and day 2. The effects of coping style, salinity and time on plasma osmolality concentration were assessed using a three-way mixed ANOVA. Once the interactions among the factors were determined, plasma osmolality and ionic concentration were compared within each coping style using one-way ANOVA with time set as within-subjects factor and salinity set as a between-subjects factor. Plasma osmolality and ionic concentration and branchial gene expression were also compared within each coping style using one-way ANOVA only with salinity set as a between-subjects factor. All one-way ANOVA tests were followed by Tukey’s HSD test. All data are expressed as mean ± SEM, and the significance level was *p* < 0.05.

## Results

### The Principal Component Analysis of the Behavioral Experiment

**Figure 1:**
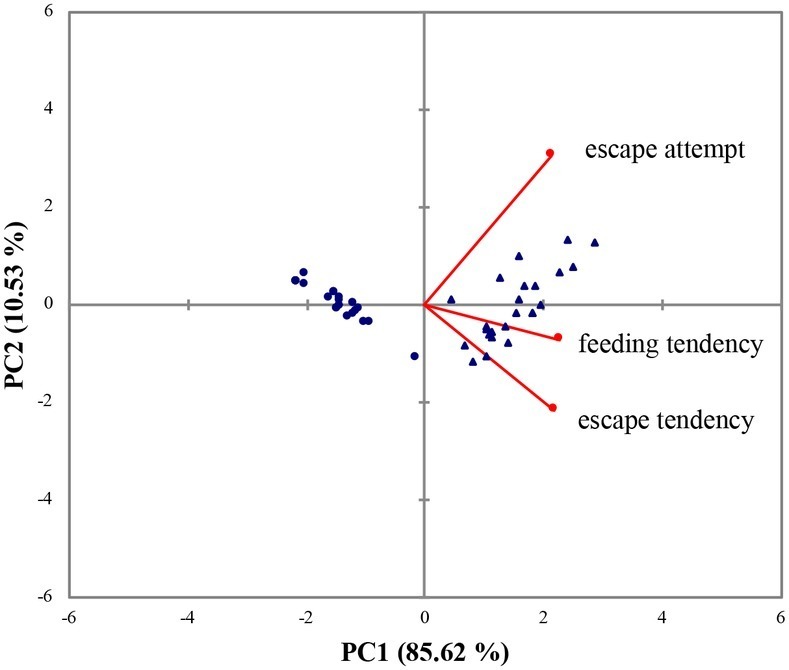
Biplot of principal component analysis (PCA) loadings scores and variables (escape attempt, feeding tendency, and escape tendency) for the flounder dataset. (▲) represents AC flounder; (●) represents PC flounder.

In the series of behavioral tests, AC and PC flounder showed a divergence in escape and feeding behavior. The AC flounder were more motivated to feed and showed a higher tendency to escape during the confinement than PC flounder. The two principal components, PC1 and PC2, explained 85.62 and 10.53% of total variance, respectively, showing a clear way to discriminate between the two coping styles. According to PCA, escape attempts, feeding tendency and escape tendency all loaded positively onto PC1 (Figure 1). The eigenvectors and correlation between variables of PC1 and PC2 are shown in Table 2.

**Table 2:** Principal component analysis; and eigenvectors and correlation between variables of two principal components PC1 and PC2.

Principal component	% Variability		Feeding tendency	Escape attempt	Escape tendency
PC1	85.62%	Eigenvectors	0.598	0.578	0.555
		Correlation	0.958	0.927	0.890
PC2	10.53%	Eigenvectors	−0.193	−0.568	0.8000
		Correlation	−0.109	−0.319	0.450

### Swimming Activity of Flounder After Transfer From Seawater to Freshwater

**Figure 2:**
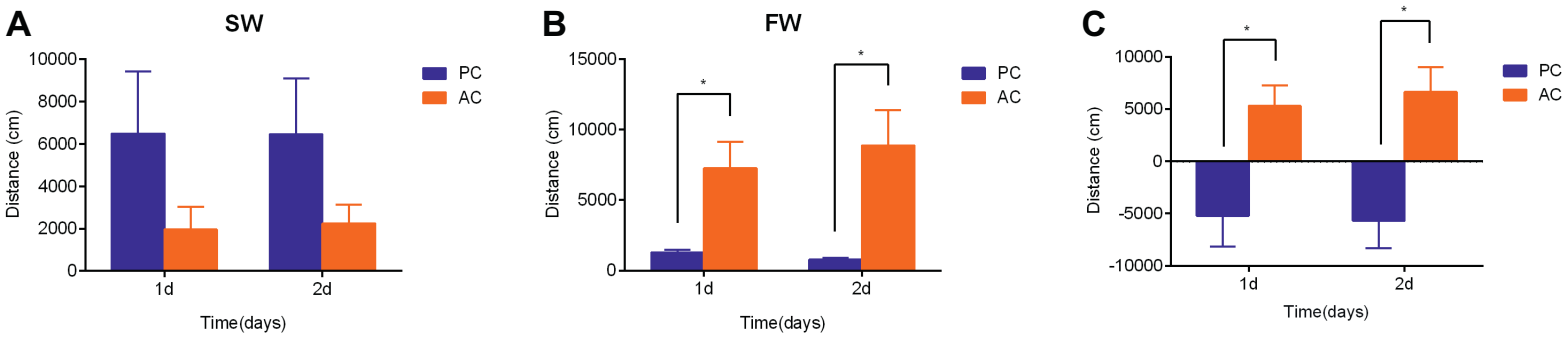
The swimming activity (i.e., distance moved in cm) of passive coping style (PC) and active coping style (AC) olive founder after transference from SW to SW **(A)** and FW **(B)**. **(C)** Summary of the direction and magnitude of difference in distance moved for PC and AC fish after transfer from SW to FW. The data in **(C)** were calculated using the values of total swimming distance of each fish in FW minus the total swimming distance of fish in SW at each time point (1 day and 2 days post transfer). Data are mean ± SEM (*n* = 8). An asterisk denotes a significant difference between the data within the same time group (*p* < 0.05).

The swimming activities of PC and AC flounder were both modified 2 days after being transferred from SW to FW. The swimming distance of PC flounder appeared higher than that of AC flounder in SW at 1 day and 2 days but no significant difference was seen between these groups (Figure 2A). However, after FW transfer, AC flounder moved significantly further than PC flounder after 1 day (ANOVA, *F* = 29.69, *p* = 0.015) and 2 days (ANOVA, *F* = 29.71, *p* = 0.014) of salinity challenge (Figure 2B). A marked significant difference in the distance moved by two coping styles was therefore seen with PC flounder significantly reducing its swimming distance and AC flounder significantly increasing its swimming distance after both 1 day (ANOVA, *F* = 0.907, *p* = 0.01) and 2 days (ANOVA, *F* = 0.266, *p* = 0.004) of FW transfer (Figure 2C). The trend for increasing distance in AC fish but decreasing distance for PC fish was revealed as a significant interactive effect between salinity and coping style at both day 1 (ANOVA, *F* = 8.4, *p* < 0.01) and day 2 (ANOVA, *F* = 10.8, *p* < 0.01).

### Plasma Osmolality

**Figure 3:**
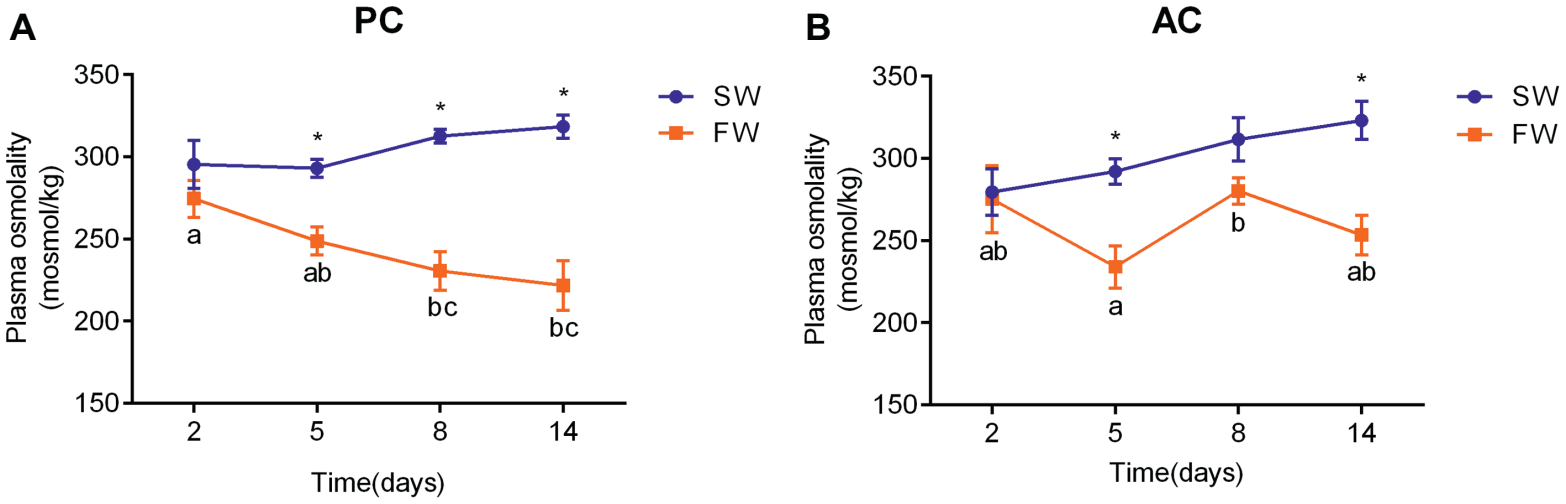
Temporal changes in the plasma osmolality of olive flounder **(A)** with passive coping style (PC) and **(B)** with active coping style (AC) with fish either remaining in seawater (the SW control, ●) or after transference to freshwater (FW, ■). Data are mean ± SEM (*n* = 6). Different letters denote a significant difference across time within each SW or FW group (*p* < 0.05). An asterisk signifies a significant difference between the SW and FW groups at each time point (*p* < 0.05).

The plasma osmolality of PC (ANOVA, *F* = 5.748, *p* < 0.001) and AC flounder (ANOVA, *F* = 0.473, *p* = 0.003) transferred to FW were lower than those of fish in SW (Figure 3). There were no significant changes observed in plasma osmolality of SW-acclimated AC and PC flounder. The plasma osmolality of PC flounder gradually declined from 2 to 14 days after transference to FW, and showed a significant difference between SW and FW at 5 days (ANOVA, *F* = 1.525, *p* = 0.002), 8 days (ANOVA, *F* = 7.593, *p* = 0.001), and 14 days (ANOVA, *F* = 5.313, p = 0.001), respectively (Figure 3A). The plasma osmolality of PC flounder decreased to below 223 mOsm at 14 days after transference to FW, and was significantly different to the osmolality of the PC freshwater fish at 2 days (ANOVA, *F* = 3.85, *p* = 0.024; Figure 3A). In contrast, the plasma osmolality of FW-transferred AC flounder did not show a consistent decline over time but instead fluctuated around an average level of 260 mOsm from 5 days with a significant difference between SW and FW only at 5 days (ANOVA, *F* = 0.978, *p* = 0.005) and 14 days (ANOVA, *F* = 0.005, *p* = 0.003; Figure 3B). The impact of coping style, time, and salinity on plasma osmolality was explained by interactions between salinity and time (ANOVA, *F* = 5.086, *p* = 0.003), and coping style and salinity (ANOVA, *F* = 5.429, *p* = 0.023). Four AC flounders began to die after FW-transfer since 8 days after transfer to FW.

### Plasma Ionic Concentration

**Figure 4:**
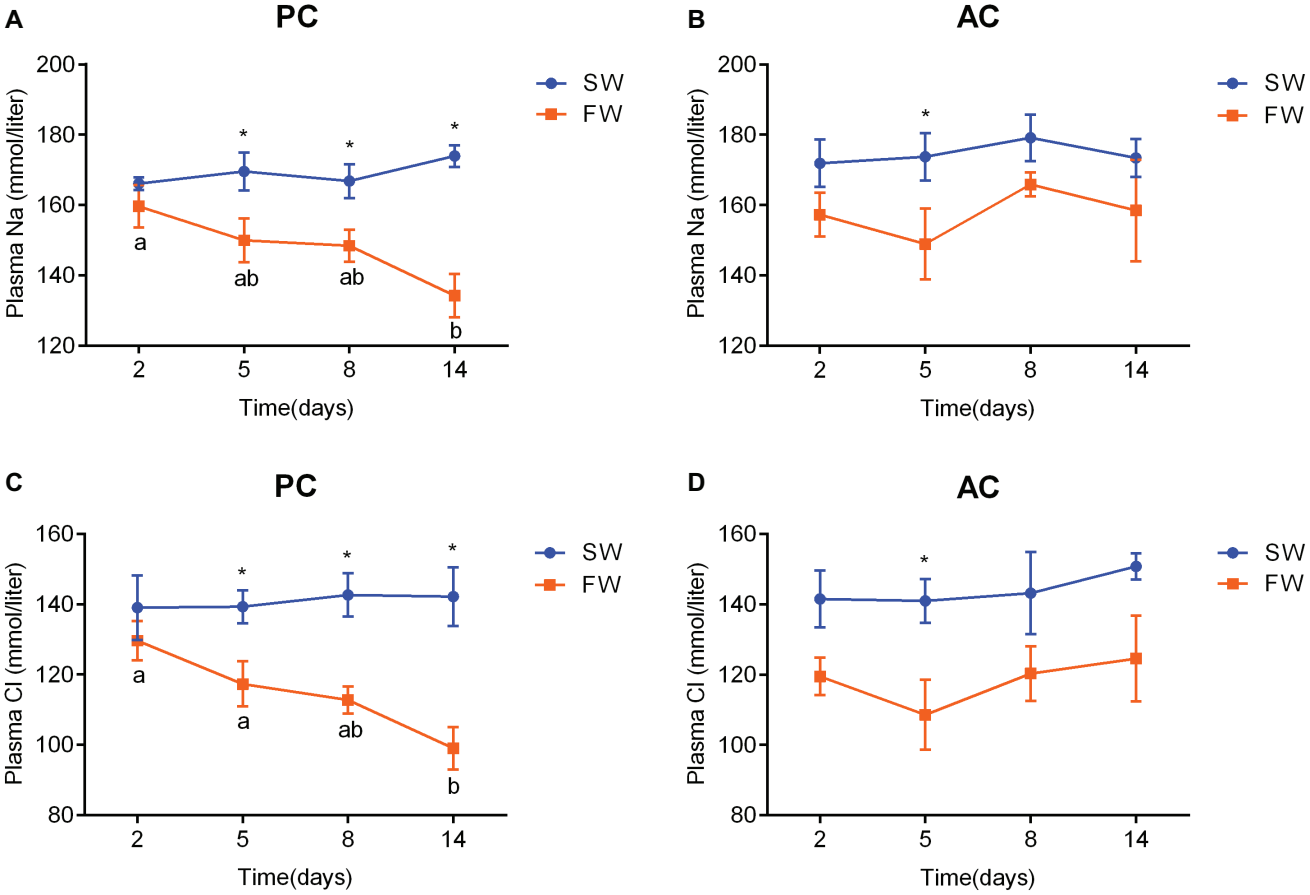
Changes in plasma Na^+^ **(A,B)** and Cl^−^ concentration **(C,D)** of olive flounder with passive coping style (PC) and active coping style (AC) either in seawater (the SW control, ●) or after transference to freshwater (FW, ■). Data are mean ± SEM (*n* = 6). Different letters denote a significant difference across time within each SW or FW group (*p* < 0.05). An asterisk signifies a significant difference between the SW and FW groups at each time point (*p* < 0.05).

Plasma Na^+^ and Cl^−^ in both PC (Na^+^, ANOVA, *F* = 2.688, *p* < 0.001; Cl^−^, ANOVA, *F* = 0.605, *p* < 0.001) and AC flounders (Na^+^, ANOVA, *F* = 1.015, *p* = 0.007; Cl^−^, ANOVA, *F* = 0.256, *p* < 0.001) were higher in SW-acclimated fish than FW-acclimated fish (Figure 4). Plasma Na^+^ and Cl^−^ of PC gradually decline from 2 to 14 days after transferred from SW to FW and a significant difference was observed between SW and FW at 5 days (Na^+^: ANOVA, *F* = 0.01, *p* = 0.048 and Cl^−^: ANOVA, *F* = 2.078, *p* = 0.026), 8 days (Na^+^: ANOVA, *F* = 0.228, *p* = 0.031 and Cl^−^: ANOVA, *F* = 0.569, *p* = 0.006), and 14 days (Na^+^: ANOVA, *F* = 1.136, *p* = 0.001 and Cl^−^: ANOVA, *F* = 0.064, *p* = 0.008), respectively (Figures 4A,C). The plasma Cl^−^ of FW-acclimated PC at 14 days was significantly different to the values of the same group at 2 days (ANOVA, *F* = 4.294, *p* = 0.003) and 5 days (ANOVA, F = 4.294, *p* = 0.044), respectively (Figure 4C), while plasma Na^+^ of FW-acclimated PC at 14 days was only significantly different to the values of the same group at 2 days (ANOVA, *F* = 3.228, *p* = 0.008; Figure 4A). In contrast, plasma Na^+^ of FW-acclimated AC flounder declined from 2 to 5 days, and elevated sharply to above 165 mmol/L at 8 days, and declined again at 14 days. There was a significant difference in AC flounder between SW and FW at 5 days (ANOVA, *F* = 0.257, *p* = 0.03; Figure 4B). The pattern of plasma Cl^−^ of AC flounder was similar to plasma Na^+^ as it declined from 2 to 5 days, and elevated at 8 days, but remained stable from 8 to 14 days after transference to FW, and a significant difference was seen at 5 days between SW and FW (ANOVA, *F* = 0.287, *p* = 0.026; Figure 4D). In contrast to the steady decline in plasma Na^+^ and Cl^−^ of PC flounder during FW-acclimation, the plasma Na^+^ and Cl^−^ of AC flounder fluctuated below the baseline level of SW-acclimated control group (Figure 4).

### Branchial Gene Expression of NKA-α1, NHE-3-like, and Rhcg

**Figure 5:**
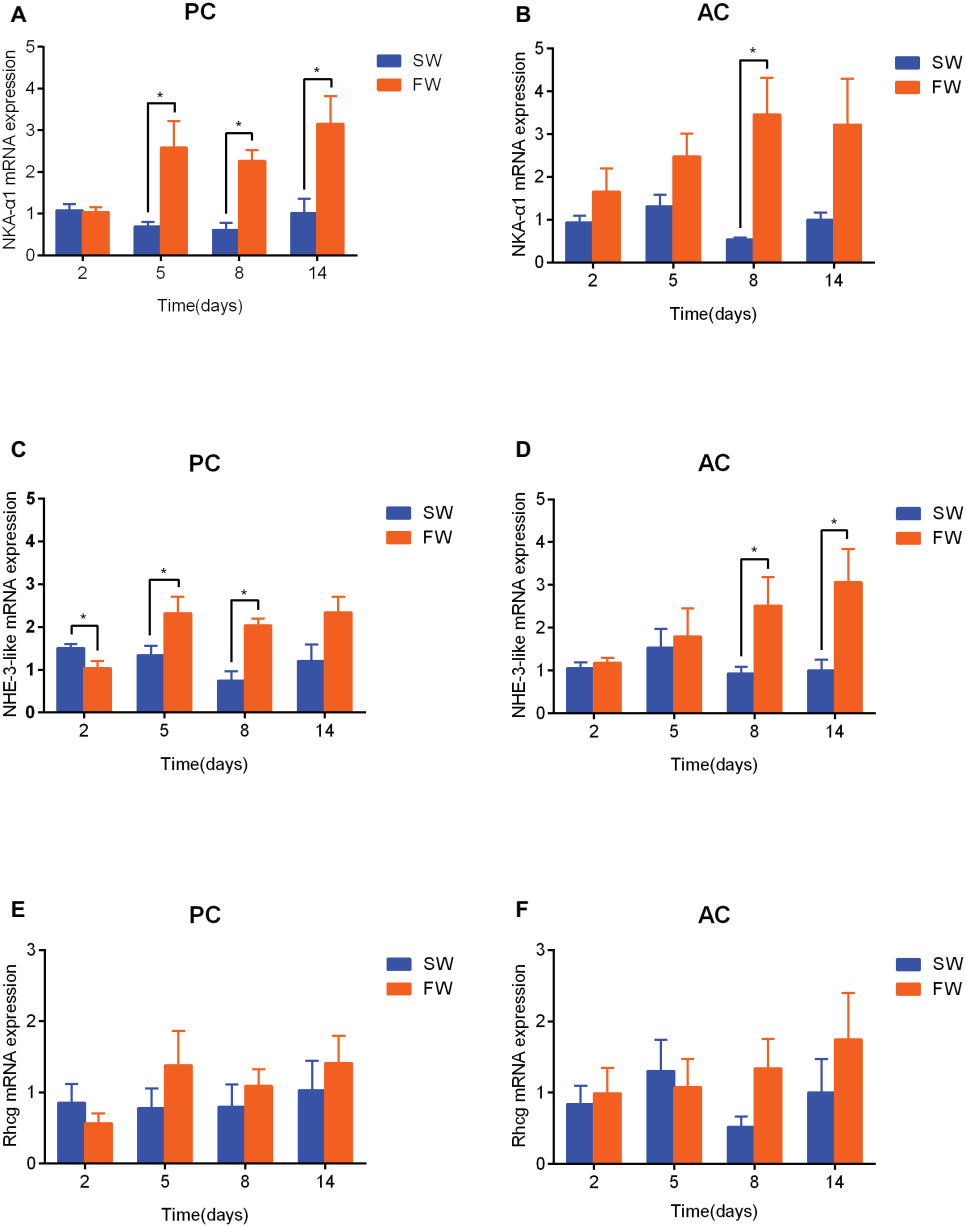
The branchial gene expression of NKA-α1 **(A,B)**, NHE-3-like **(C,D)** and Rhcg **(E,F)** in passive coping style (PC) and active coping style (AC) of olive flounder after transfer from seawater (SW) to freshwater (FW). Data are mean ± SEM (*n* = 6). An asterisk signifies a significant difference between the SW and FW groups at each time point (*p* < 0.05).

In comparison with the time matched control group, the expression of NKA-α1 mRNA in PC flounder significantly increased from 5 to 14 days (5 days: ANOVA, *F* = 12.209, *p* = 0.029; 8 days: ANOVA, *F* = 1.23, *p* < 0.001, and 14 days: ANOVA, *F* = 7.403, *p* = 0.022; Figure 5A), while the NKA-α1 expression of AC flounder only significantly increased at 8 days (ANOVA, *F* = 9.535, *p* = 0.02; Figure 5B). Again compared to the time matched control, the expression of NHE-3-like mRNA in PC flounder was significantly decreased at 2 days (ANOVA, *F* = 1.939, *p* = 0.033), but remarkably increased at 5 days (ANOVA, *F* = 0.49, *p* = 0.049) and 8 days (ANOVA, *F* = 0.117, *p* = 0.001) (Figure 5C). In AC flounder, the NHE-3-like expression of FW-acclimated flounder was significantly increased at 8 days (ANOVA, *F* = 8.548, *p* = 0.042) and 14 days (ANOVA, *F* = 1.618, *p* = 0.046) compared with the SW-acclimated controls (Figure 5D). The expression of Rhcg mRNA in both of PC and AC flounders observed no significant differences between SW and FW (Figures 5E,F). Interestingly, in similarity with the expression profile of NHE-3-like gene, Rhcg expression in FW-acclimated PC flounder tended to be lower than that of SW group at 2 days and slightly higher from 5 to 14 days, but without significant difference (Figure 5E), while Rhcg expression of FW-acclimated AC flounder tended to be higher than that of SW group at 8 and 14 days, but there was no significant difference (Figure 5F).

### Schematic Model for Coping Strategies in Passive Coping and Active Coping Flounder Under Salinity Challenge

**Figure 6:**
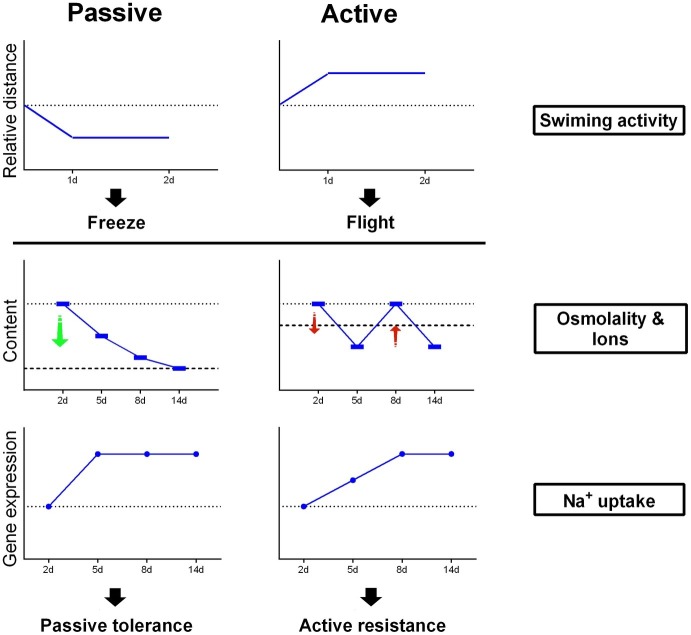
A schematic outline of coping strategies for PC and AC flounder undergoing salinity challenge.

As shown in Figure 6, PC flounder reduced swimming activity after transfer from SW to FW within 2 days, while AC flounder showed an increase in locomotion. For osmolality and ionic content of flounder plasma, FW-acclimated PC flounder show a constant decline in their physiological profile but FW-acclimated AC flounder show a fluctuating level compared to the physiological baseline of control fish in SW. According to gene expression related to Na^+^ uptake, the gene expression of PC flounder increases sharply at 2 days after transfer to FW while the gene expression of AC flounder reveal a more delayed response, only showing a modest increase from 2 to 8 days after transfer.

## Discussion

Our study demonstrates marked differences in the behavioral and physiological coping strategies of PC and AC olive flounder in response to salinity challenge. The AC flounder showed higher feeding tendency and more willingness to escape under net confinement than PC flounder. AC individuals also adopt an “active resistance” defense strategy to retain plasma composition during hypoosmotic challenge, which is underpinned by initially increasing swimming activity level to escape and gradually increasing the expression of branchial osmoregulatory genes for hypoosmotic adaptation. Conversely, PC individuals appear to employ a “passive tolerance” strategy with a decrease in the plasma osmolality and ionic content, underpinned by a decrease in swimming activity level as well as a sharp increase in the expression of branchial osmoregulatory genes including NKA-α1, NHE-3-like, and Rhcg. This is therefore the first study showing that coping strategies influence the way fish deal with salinity challenge.

Previous studies suggest that passive and active individuals respond differently to novel situations or a change in the environment and a number of different parameters can be recorded to quantify this difference in coping style. For example, the time taken to resume feeding in a novel environment, or after stress, has become the most useful and informative ways of assessing different coping styles in fish (Overli et al., 2006, 2007; Kittilsen et al., 2009; Silva et al., 2010; Martins et al., 2011c; Basic et al., 2012). Indeed, fish that resume feeding faster after transfer to a novel environment are typically ascribed to being active individuals (Martins et al., 2011a; Rupia et al., 2016). But other parameters are valuable too because the time taken to react to the presence of food or time taken to avoid stressors have also been used to assess coping style in animals (Brelin et al., 2005; Silva et al., 2010; Laursen et al., 2011; Martins et al., 2011d). Many different parameters can therefore be used so propensity to feed and air exposure has been used in this study to distinguish behavioral coping styles. According to behavioral tests, the results of the PCA indicated that AC and PC flounders were divergent in the way they responded to food and net confinement. AC flounder were more active and exhibited a higher tendency to feed after isolation than PC flounder. Similar results have also been seen in wild-caught olive flounder *P. olivaceus* (Rupia et al., 2016). Likewise, active dominant Nile tilapia *Oreochromis niloticus* and Atlantic salmon *Salmo salar* have a strong appetite and are more successful in competitive feeding interactions compared to passive subordinate individuals of the same species (Fernandes and Volpato, 1993; Metcalfe, 1994). A number of studies therefore suggest that active fish had an actively escape to stressor and have high feeding motivation and these are supportive of the results from our own study (Brelin et al., 2005; Silva et al., 2010; Laursen et al., 2011; Martins et al., 2011b).

Bimodality in the distribution of behavioral or physiological traits or both is not uncommon in animal populations and has been documented in insects (Riechert and Jones, 2008), fish (Brown and Bibost, 2014), birds (Favati et al., 2014), and mammals (Oortmerssen and Busser, 1989). Locomotor activity is among the commonly measured behaviors in animal personality research (Le Galliard et al., 2013). In our study, both AC and PC flounder exhibited behavioral changes after transference from SW to FW within 2 days but nature of change differed between the two coping style types. PC flounder exhibited a significant reduction in swimming activity while AC flounder significantly increased locomotion after transference during 2 days of recording. Previous studies demonstrate that active individuals employ a proactive defense response, such as flight or fight, while passive individuals employ a reactive defense response, such as freeze or hide when startled (Rupia et al., 2016). In general, the ability of animal to alter their behavior provides an adaptive advantage when facing a challenge (Dall et al., 2004) because escaping from stressors is facilitated. Moreover, active animals are willing to investigate novel environments, while passive animals are more reclusive when faced with unfamiliar situations (Overli et al., 2007; Oswald et al., 2012). Accordingly, we suggest that PC individuals faced with a hypoosmotic environment reduce their activity levels and therefore adopt a passive less energetically expensive form of “freeze” behavior, to conserve energy for FW acclimation (Brown et al., 1993). On the other hand, AC individuals adopt a proactive “flight” strategy where they increase swimming activity levels that presumably allow them to investigate a novel environment and escape. However, some AC flounders maintained higher swimming activity and began to die 8 days after transfer to FW (data not shown). These phenomena were similar to the rainbow trout *Oncorhynchus mykiss* which were exposed to severe hypoxia; non-surviving fish displayed strenuous avoidance behavior involving burst type activity while surviving fish did not panic and remained quiet (van Raaij et al., 1996). Some researchers considered that individual variation exists in the threshold for when a stimulus becomes inhibitory or stimulatory whereby coping style is linked with the subjective experience of that stimulus in a particular situation (Martins et al., 2011a). Accordingly, AC flounder are willing to escape when faced a hypoosmotic environment, when this stimulus could not become inhibitory signals in cultured condition, finally caused energy exhaustion and become distress. The success of both coping styles might therefore depend upon the variability or stability of the environment (Koolhaas et al., 1999). But only a few studies have addressed the survival value of distinct active and passive coping styles. Notably, unpublished studies in feral populations of birds indicate that the fitness of different coping styles depends on the stability of the environment in terms of social structure and food availability (Koolhaas et al., 1999). Thus, behavioral coping strategies under environmental stress appear to be highly related to the chance of survival.

When faced with a hypoosmotic environment, the plasma osmolality and ionic levels of marine fish remain stable or decline slightly (Kelly et al., 1999; Motohashi et al., 2009), while euryhaline fish significantly change plasma conditions for freshwater adaption (Evans, 2008; Kang et al., 2008). As euryhaline fish, flounder also reduce their plasma osmolality and plasma Na^+^ and Cl^−^ (which are the dominant ions in plasma) content during SW to FW-adaption (Evans, 2008; Yuan et al., 2017). In this study, differential and dynamic changes in plasma osmolality and ionic content of olive flounder with active or passive coping styles (AC and PC) were observed during FW acclimation. The plasma osmolality of FW-acclimated PC flounder gradually declined to below 223 mOsm at 14 days, while it fluctuated around 260 mOsm in FW-acclimated AC flounder. The pattern of plasma osmolality and ionic content of FW-acclimated PC flounder showed a significant and lasting decrease during FW-acclimation and is similar to that seen in other euryhaline teleosts such as brackish medaka *Oryzias dancena* (Kang et al., 2008) and European sea bass *Dicentrarchus labrax* (Jensen et al., 1998). In contrast, the pattern of plasma parameters in FW-acclimated AC flounder showed a fluctuation under the baseline level of the control group and is similar to the response seen in marine fish. Such results indicate that markedly different iono- and osmoregulatory processes exist between AC and PC flounders. Previous studies suggest that subordinate rainbow trout *Oncorhynchus mykiss* display higher Na^+^ uptake and excretion than dominant fish under social stress (Sloman et al., 2002, 2004), with the assumption that chronic stress impacts the ionoregulatory ability of subordinate trout in a negative way. Indeed, that subordination stress results in an increased branchial efflux of sodium suggests that the ionoregulatory ability of subordinate trout is compromised (Sloman et al., 2004). Therefore, on one hand, it could be considered that PC individuals have a compromised ionoregulation based on the higher ion efflux rate compared to AC fish. Indeed, the salinity stress associated with PC flounder appeared to result in an increased branchial efflux of Na^+^, driving the need for higher uptake of Na^+^ across the gills and then increasing the expression of branchial Na^+^-related gene. In contrast, it would appear that AC flounder adopt an “active resistance” defense strategy by maintaining original plasma conditions. However, on the other hand, the metabolic performance of *P. olivaceus* with active coping style or passive coping style appears to be constant between SW and FW (Rupia et al., 2016), indicating that the energy consumption of olive flounder secreting NaCl in seawater or extracting NaCl in freshwater is approximately the same. Iono- and osmoregulatory processes are achieved by various enzymes and transporters, and the synthesis and operation of these transport related proteins is energetically expensive (Tseng and Hwang, 2008). Boeuf and Payan theorized that osmoregulation costs 20–68% of the total energy expenditure in different species (Bœuf and Payan, 2001). Active individuals, however, have a prominently higher standard metabolic rate (SMR), maximum metabolic rate (MMR), and absolute aerobic scope (AAS) than passive individuals (Rupia et al., 2016) meaning that AC individuals can potentially accommodate metabolic costs more easily. Therefore, when faced with a hypoosmotic environment, AC flounder are possibly capable of spending more energy on escape and iono- and osmoregulation and thus performed higher levels of swimming activity while simultaneously maintaining original plasma osmolality and ionic concentrations. In contrast, as a result of a lower metabolic space, PC flounder were possibly forced to reduce their swimming activity and conserve energy in FW adaption with a less energetically demanding decline in plasma osmolality and ionic content.

“FW-type” ionocytes of killifish *Fundulus heteroclitus* located with NKA and NHE3-like, are responsible for NaCl uptake and appear in the fish gill after transfer from SW to FW (Hwang and Lee, 2007; Takei et al., 2014). NKA, NHE3, and Rhcg are also both involved with “NHE3 cells,” which is a major type of ionocytes (over 90%) in FW-acclimated medaka *Oryzias latipes* (Lin et al., 2012) and are responsible for Na^+^ uptake, acid secretion and NH_4_^+^ excretion (Hsu et al., 2014). NKA creates an electrochemical gradient to drive ions (like Na^+^ and Cl^−^) actively across the branchial epithelia to maintain intracellular homeostasis in teleosts (Hwang et al., 2011). It had also been demonstrated that salinity challenge would change NKA activity as well as the abundance of NKA-α-subunit mRNA in fishes (Scott et al., 2004; Hwang and Lee, 2007; Moorman et al., 2014). Previous studies found that sea bass *Dicentrarchus labrax* (Varsamos et al., 2002), milkfish *Chanos chanos* (Lin et al., 2003; Hwang and Lee, 2007) and brackish medaka *Oryzias dancena* (Kang et al., 2008) both increased the NKA activity after FW acclimation. This study confirmed that branchial expression of NKAα1 was up-regulated in both AC and PC flounders during FW-acclimation. On the other hand, NHE as a transporter to compensate Na^+^ uptake has been well established (Shih et al., 2012; Chang et al., 2013; Guh et al., 2015). More recent studies demonstrated that branchial NHE expression levels and Na^+^ uptake were both upregulated by low-Na^+^ FW acclimation in tilapia *Oreochromis mossambicus* (Inokuchi et al., 2009), medaka *Oryzias latipes* (Wu et al., 2010) and zebrafish *Danio rerio* (Lin et al., 2008). Similarly, branchial Rhcg1 expression was also induced by low-Na^+^ FW acclimation in medaka *Oryzias latipes* (Wu et al., 2010). In flounder, our study indicated that the branchial NHE-3-like and Rhcg mRNA expression of AC and PC were both up-regulated in response to FW adaption. Further evidence suggests that NHE3 and Rhcg1 were involved in fish ammonium-dependent Na^+^ uptake model (Hwang et al., 2011). Thus, NKA-α1, NHE3-like, and Rchg are all suggested to play critical roles in sodium uptake in FW-acclimated olive flounder. However, notably, AC flounders delayed these sodium uptake related gene expression than PC flounders. Indeed, the gene expression of PC flounders increased sharply at 2 days after transference from SW to FW, while the gene expressions of AC flounders only increased modestly from 2 to 8 days after transference. Accordingly, such differences indicated that: AC flounders gradually increased the expression of Na^+^ uptake related genes (i.e., NKAα1, NHE3, and Rhcg), and was accompanied by an up-regulation of acid secretion and ammonia excretion and also enhanced the function of “NHE3 cell like” or “FW-type like” ionocytes for adaption to a hypoosmotic environment. In contrast, PC flounder show a sharply increase in these gene expressions after transfer to FW. However, a previous study has reported that SW-acclimated active flounder show a higher expression level of NKCC1 compared to passive flounder (Zou et al., 2019). NKCC, a major secretory ion transporter, is located on the basolateral side of ionocytes and is responsible for NaCl secretion in most SW-acclimated euryhaline teleost (Kang et al., 2010; Hwang et al., 2011). Moreover, NKCC is reported to play a role in Na^+^/Cl^−^ uptake of FW-acclimated tilapia (Hiroi et al., 2005; Hwang and Lee, 2007) and other studies propose that NKCC may also be involved in ammonia dependent Na^+^ uptake by transporting the basolateral NH_4_^+^ (Hiroi and McCormick, 2012; Wright and Wood, 2012). Thus, due to the higher expression and reserve level of NKCC seen in AC flounder in seawater, it is suggested that AC flounder gradually increase Na^+^ uptake related gene expression when faced with FW while PC flounder sharply increase these gene expression and quickly increase sodium uptake from ambient water environment. Consequently, when considering gene expression and plasma parameters collectively, PC flounder appear to adopt a “passive tolerance” strategy in their adaptation to hypoosmotic environments because plasma osmolality and plasma ionic content decreases while the expression of Na^+^ uptake related osmoregulatory genes (i.e., NKA-α1, NHE-3-like and Rhcg) increases quickly. In contrast, AC flounder retain original plasma conditions, they gradually increase the expression of NKA-α1, NHE-3-like and Rhcg, adopt a more “active resistance” defense strategy in response to salinity challenge.

## Conclusion

Our results demonstrate clear differences in the way that AC and PC flounders respond to acute stress. The AC flounder showed higher feeding tendency and more willing to escape under net confinement than PC flounder. AC individuals adopt “active resistance” defense strategy to retain the plasma composition during hypoosmotic challenge, which was underpinned by an initially increasing swimming activity level to escape and a gradually increasing level of osmoregulatory gene expression in the gills for hypoosmotic adaptation. Conversely, PC individuals employed a “passive tolerance” strategy with decreasing the plasma osmolality and ionic content, which was underpinned by decreasing swimming activity level and a sharp increase in the expression of branchial osmoregulatory gene, including NKA-α1, NHE-3-like and Rhcg, to conserve energy with the osmoregulatory process. This study clearly indicates that olive flounder with active or passive coping styles adopt different coping strategies in respond to salinity challenge which are “flight-active resistance” and “freeze-passive tolerance,” respectively. In the context of heavy rainfall and flooding, fish encounter salinity challenge for extended periods of time, and adopting different coping strategies may affect their vulnerability and mortality. This detailed insight into the coping strategies of fish improves our understanding of individual variability and welfare may therefore contribute to improved sustainability of the aquaculture industry. In addition, as the global average surface temperature increase, causing ocean warming, future studies should consider the long-term effects of wide temperature range associated with coping style for this species.

## Data Availability Statement

The datasets generated for this study can be found in the figshare: https://figshare.com/articles/Original_Data_xlsx/9987797.

## Ethics Statement

The animal study was reviewed and approved by The Animal Ethics Committee of Shanghai Ocean University (Shanghai, China). Animal procedures abide by the Guidelines on Ethical Treatment of Experimental Animals established by the Ministry of Science and Technology, China.

## Author Contributions

JZ, NH, and WL designed experiments and wrote the manuscript. JZ carried out experiments and analyzed experimental results.

### Conflict of Interest

The authors declare that the research was conducted in the absence of any commercial or financial relationships that could be construed as a potential conflict of interest.
